# PLANNING FOR AGING IN PLACE IN RURAL AREAS: PERCEPTIONS AND NEEDS OF OLDER ADULTS

**DOI:** 10.1093/geroni/igz038.945

**Published:** 2019-11-08

**Authors:** Raven H Weaver, Cory Bolkan

**Affiliations:** 1 Washington State University, Pullman, Washington, United States; 2 Washington State University, Vancouver, Washington, United States

## Abstract

Most individuals prefer to live independently in their homes, but will need support to age-in-place safely. Rural-dwelling individuals historically have worse health, limited income, and restricted access to adequate services/supports compared to their urban counterparts. Community-based aging services organizations (i.e., Area Agency on Aging; AAA) offer in-home health, social support, and information/referral to community resources that support older adults in both urban and rural communities. A representative sample of adults aged 60+ (N=253, mean age=74) were surveyed via computer-assisted-telephone interviews about their health status, needs, and service utilization. Over half (54%) lived in rural counties, which was significantly associated with receiving insufficient health care services (X2=9.227, p=.002). Insufficient service access was also associated with experiencing a fall (X2=7.315, p=.007). While 53% reported having chronic conditions, most individuals still reported good health and their top reported needs included: yard work, interior/exterior house repairs, and housework. Content analysis of open-ended survey responses regarding future care needs revealed participants anticipate help from family/friends or neighbors; reliance on physicians for referrals; and expect insurance to cover their needs. Participants had varying awareness levels of available community resources and identified concerns about adequacy of services (e.g., mental health; transportation) and health insurance barriers (e.g., reimbursement; vision/dental coverage). Preparing for future needs and anticipating changing functional capacity is critical, especially among rural-dwelling older adults with chronic conditions. To improve ability for adults with diverse needs to age-in-place, preventive services/supports that span the continuum of care needs and that complement informal family care are necessary.

